# Supplementary material for: Are carbon emissions trading and green financial instruments synergistic? -Comprehensive quantitative research based on content analysis
Source: PLoS One. 2024 Mar 7;19(3):e0298601. doi: 10.1371/journal.pone.0298601 (PMC10919601; doi:10.1371/journal.pone.0298601)
Supplement: S1 Data — (ZIP) [file pone.0298601.s001.zip › Data/地级市Z_F工作报告.zip!/║╙▒▒/║Γ╦«╩╨/└┤╫╘╣1⁄2╓┌║┼í╛┬φ┐╦╩2╛▌═°í┐_2016.docx]

各位代表：

从今年开始，我们已进入第十三个五年规划时期。根据党的十八届五中全会、省委八届十二次全会和市委三届七次全会精神，衡水市政府编制了《衡水市国民经济和社会发展第十三个五年规划纲要(草案)》。现在，我代表衡水市人民政府向大会作工作报告，请各位代表连同《纲要(草案)》一并审议，并请市政协委员和列席会议的同志提出意见。

一、“十二五”时期经济社会发展回顾

过去五年，在中共衡水市委坚强领导下，全市上下深入学习贯彻习近平总书记系列重要讲话精神，认真落实党中央、国务院和省委、省政府决策部署，紧紧围绕 “强势开局、跨越赶超，全面建成小康社会”总要求，坚持稳中求进工作总基调，全力以赴抓改革、打基础、造环境、铸优势、求赶超，胜利完成“十二五”规划主要目标任务，在全面建成小康社会进程中迈出坚实步伐。

坚持发展第一要务，全力以赴抓投资、稳增长。累计完成固定资产投资4187亿元，是“十一五”的2.7倍。实施省、市重点项目532个，完成投资 2224.3亿元，其中战略性新兴产业、装备制造业和服务业项目占比超过70%。新上亿元以上项目1900多个，海伟丙烷脱氢、阳煤乙二醇项目投资均超百亿元，泰纳改性合成橡胶新材料、老白干酿酒机械化改造、瑞丰精密铸造、蒙牛高端奶、澳泰环保制冷压缩机组等234个项目投产或部分投产，泰华服务机器人、亚森工业机器人、通广民用航空通导装备、安德森智能家居、冀中能源矿用智能装备等重大项目开工建设。基础设施建设力度加大。大广高速、邯黄铁路、邢衡高速衡水段一期、衡德高速故城支线、衡井线建成通车，石济客专、邢衡高速衡水段二期、106国道衡水湖段改线开工建设。近两年交通、水利年度完成投资均超百亿元。项目谋划跑办水平有了新提高。入库重大储备项目373项，总投资6747亿元，实现了由“来了政策机遇没项目”到“瞄着政策谋项目、带着项目跑政策” 的转变。石衡高速即将开工，京衡客专项目已启动路线方案研究，衡水机场前期跑办取得重大进展。

把转方式、调结构放在突出位置，着力提高发展质量和效益。三次产业比重由19.7:50.7:29.6调整到13.8:46.2:40。现代农业强市建设扎实推进。粮食总产稳定在35亿公斤以上，畜牧、蔬菜、果品三大产业稳步提升，规模农业、法人农业、科技农业不断壮大，建成国家级现代农业示范区1 家，省级现代农业园区3家，农业产业化经营率达到66.8%。工业转型升级迈出坚实步伐。确立“6+3”主导产业发展方向，实施“两改造一倍增”“1+10”行动计划和现代企业制度建设、企业家素质提升、企业整合重组三大工程。工业投资年均增长22.6%，其中技改投资增长25.1%。超半数规模以上工业企业建立现代企业制度。年主营业务收入超200亿元产业达到3个，规模以上工业企业新增588家达到1234家，年纳税超千万元企业由44家增加到194家，养元公司年主营业务收入突破百亿元、纳税超10亿元。大众创业氛围日益浓厚，市场主体由9.83万户增加到19.42万户。万众创新扎实开展，认定省、市级众创空间13家，科技型中小企业和科技小巨人企业分别达到2200家、160家。现代服务业加快发展。电子商务交易额年均增长30%以上，玛世公司被评为国家级电子商务示范企业，大营裘皮电子商务基地被列为省级电子商务示范基地。安平国际丝网物流聚集区、衡德物流园晋升省级物流产业聚集区。旅游总收入较“十一五”增长3.75倍，衡水湖景区、武强年画博物馆晋升4A级景区，衡水湖景区成为全省唯一的国家生态旅游示范区。实施文化产业“一县一品”和“龙头带动”工程，周窝音乐小镇、闾里汉文化民俗小镇知名度不断扩大，金音乐器集团、习三内画公司成功创建国家级文化产业示范基地。

统筹城乡发展，加快城镇和美丽乡村建设步伐，城乡面貌发生重大变化。确立近期“一湖三区”、远期“一湖六区”大衡水格局和城市组团式发展思路，设立滨湖新区，整合工业新区，改造河东旧城区，建设南部新城区，拉大城市框架。市区完成26条主干街道、10座桥梁建设，改造提升小街巷185条，前进大街全线通车，榕花大街成为连接三区、贯通南北的重要通道，全长123公里的高速外环即将完工。马拉松广场、龙源国际、泰华雅阁、衡百国际、怡然城、哈院综合楼、衡水学院新校区、衡水第一中学、职业教育园区等城市功能性项目投入运行。实施主干街道地下管网改造、地下垃圾池取缔、便道砖铺装、天然气路北环网、集中供热一次网互联等工程，城市功能日益完善，品位不断提高。组织开展地下管线普查和信息系统建设，建成全国首批、全省首个“全光网城市”。以提质扩容为重点，加强县城建设。县城建成区总面积达136.7平方公里，年均增长8.4%，7个县市建成省级园林县城。扎实推进美丽乡村建设。以衡水湖省级片区为重点，对 520个村实施改造提升，涌现出绳头庄、周家窝等升级版现代新农村。加快“三区同建”步伐，5万平方米以上联建社区达到46个，16个省级中心村开工建设。

强力推进改革开放，内生动力活力加速释放。坚持开放引领发展、改革破解难题，综合配套改革积极稳妥推进，省、市重点改革试点项目全部启动。在全省率先实施工商营业执照、机构代码证、税务登记证“三证合一”，全面实施“一照一码”登记制度改革。市、县、乡机构改革基本完成。全市首个行政审批局在工业新区挂牌成立。农业农村改革始终走在全省前列，率先整市推进农村土地承包经营权确权登记工作，已完成确权登记807万亩。土地流转面积323.2万亩。 3000亩以上规模经营典型78个，注册家庭农场4978家，登记农民合作社12527家，总量均居全省首位。深化户籍制度改革，实施省内跨市户口迁移 “一站式”办理。探索金融创新改革，上市企业由2家增加到27家，交通银行、民生银行、浦发银行、河北银行、邢台银行等金融机构入驻我市，连续三年被省政府评为“金融生态市”。抢抓京津冀协同发展重大机遇，确立了“一枢纽四基地”功能定位，深入推进“三个一百”工程，“蓝火计划”衡水基地正式挂牌。威克多制衣、太阳大地公司等一批京津企业落户我市。“衡水湖号”客运专列顺利开通。衡水海关开关运行。成功举办四届衡水湖国际商务洽谈会，签约项目150个，总投资2031亿元。

大力实施地下水超采综合治理和植树造林，铁腕治理大气污染，着力放大生态优势。单位生产总值能耗累计降低20%以上，化学需氧量、氨氮、氮氧、二氧化硫等主要污染物减排均完成省下达任务。开展“环保攻坚年”行动，实施大气污染防治行动计划和一揽子政策，以前所未有的举措推进环境治理工作，一大批危害群众健康和环境安全的问题得到整治。圆满完成“APEC蓝”“阅兵蓝”空气质量保障任务。市区344辆环保型公交车上线运营，淘汰黄标车46136辆，完成裸露土地绿化硬化50万平方米，卫生保洁机械化清扫率由23%提高到80%。全面取缔砖瓦窑223座。空气质量综合指数较2013年下降18.85%，达标天数由70天增加到120天。市、县城区污水和垃圾处理体系基本建成，逐步向重点乡镇和农村延伸。地下水超采综合治理完成投资62亿元，实现地下水压采能力5.82亿立方米。南水北调中线配套工程基本完工。提前一年实现“一人一亩林”目标，衡水湖湿地公园、冀州小寨森林公园等10个林业生态观光项目单体规模超万亩。衡水湖被列入全国水质较好江河湖泊生态环境保护支持范围，自然保护区范围和功能区划调整获国务院批准。

努力保障和改善民生，切实让人民群众得到实惠。坚持每年办一批惠民好事实事，民生支出占财政支出的79%，由2010年的83.7亿元增加到222.2亿元。农村居民人均可支配收入达到9030元，比2010年翻了一番;城镇居民人均可支配收入由14525元增加到21615元。城镇累计新增就业19.8万人。社会保障体系更加健全，城镇、农村低保标准分别比2010年提高 74.1%、84.2%，城镇社区居家养老服务中心、农村互助幸福院覆盖率分别达到90%、66%，市、县财政支出3700多万元，为全市80471名 80岁以上老人发放了高龄补贴。开工建设保障性住房7.79万套，城镇低收入住房困难家庭实现应保尽保。脱贫攻坚成效明显，41.8万贫困人口稳定脱贫，近200万农村人口饮水安全得到保障。全面实行城乡免费义务教育，主城区新扩建中小学13所，班容量过大问题得到缓解。衡水中学、衡水二中等普通高中教育水平不断提升，高等教育稳步发展，市直中等职业学校整合完成。大力实施文化惠民工程，加快各县市区“两馆一站一书屋”建设步伐，建成县级数字影院11家。衡水中国书画博物馆入选中国十大民营博物馆。丹青钜迹·中国宋元绘画(衡水)国际学术研讨会在我市举行。稳步推进医药卫生体制改革，县、乡、村全面落实国家基本药物制度。新型农村合作医疗和城镇居民医保财政补助标准由人均120元提高到380元，城乡居民大病保险实现全覆盖。衡水四院晋升三级医院。群众体育蓬勃发展，长跑城、健步城、篮球城创建成效显著。成功举办四届衡水湖国际马拉松赛并承办两届全国马拉松锦标赛。平安衡水建设扎实推进，安全生产形势总体平稳，食品药品安全监管力度不断加大，治安、消防和交管技术装备水平显著提升，重拳打击“两抢一盗”和“菜霸”“沙霸”等违法犯罪行为，社会大局和谐稳定。国防动员和双拥共建深入开展，军政军民团结局面进一步巩固。人口计生、民族宗教、新闻广电出版、外事侨务、涉台事务、人民防空、气象、防震减灾、地理信息、邮政通信、援疆、妇女儿童、老龄、残疾人、史志、档案等工作都取得新成绩。

从严从实改进作风，政府自身建设不断加强。深入推进简政放权，全部取消非行政许可审批事项，市本级行政审批事项取消下放621项。政务服务中心平台建设逐步完善，“四个清单”制度全面建立。大力推进“三级平台”“两个代办”，在全市118个乡镇4038个村建立了便民服务站点。加强廉政建设，严格落实 “八项规定”，坚决纠正“四风”，全面落实主体责任。推进依法行政，建立常务会学法制度。自觉接受人大、政协监督，人大代表建议、政协提案办结率均达到 100%。不断完善政府决策程序和工作机制，建立法律顾问制度，政府决策科学化、民主化水平不断提高。深入开展党的群众路线教育实践活动、“三严三实”专题教育和解放思想大讨论，强化担当意识，加强政策执行督查，政府行政效能进一步提升。拓宽群众诉求渠道，开通961890群众服务热线，累计解决群众反映问题16万余件。

刚刚过去的2015年，我们按照“解放思想、抢抓机遇、奋发作为、协同发展”的要求，统筹把握稳增长、调结构、促改革、治污染、惠民生、防风险的平衡点，加强投资和项目建设，全市200项省、市重点项目提前完成投资任务;加快产业转型升级步伐，技改投资和高新技术产业投资分别增长12.9%、17.1%，均居全省前列;强力治理大气污染，深入实施“净大气、抑扬尘、确保蓝天蓝”专项行动;精准推进协同发展，成功举办“京津百家纺织名企进衡水”“承接京津产业转移项目推介会”等活动;全面深化改革开放，开展“1+1”改革创新活动;坚持不懈改善民生，市委、市政府承诺的12件民生实事全面完成。全市经济继续保持增速领先、总量进位的良好态势。地区生产总值增长7.6%，规模以上工业增加值增长5%，全部财政收入、一般公共预算收入分别增长9.9%、11%，固定资产投资增长14.8%，社会消费品零售总额增长9.7%，城乡居民人均可支配收入分别增长10.2%、11.4%，居民存款余额1714亿元，城镇新增就业4万人，城镇登记失业率控制在4.5%以内，居民消费价格指数上涨1% 以内。2015年主要目标的完成，标志着我市“十二五”规划圆满收官。

各位代表!过去五年，是我市积极应对下行压力、经济加速发展、综合实力快速提升的五年，是突破基础瓶颈制约、城乡面貌发生重大变化的五年，是社会和谐稳定、人民群众得到更多实惠的五年，也是改革开放全面发力、发展方式加速转变、经济结构深刻调整、质量效益稳步提高的五年。突出表现在五个转变：

经济发展逆势上扬，实现了由总量落后向增比进位转变。主要经济指标增速位居全省前列，地区生产总值、固定资产投资跃上千亿元台阶，规模以上工业增加值、规模以上工业利润、固定资产投资、社会消费品零售总额、实际利用外资、外商直接投资、城乡居民储蓄存款7项指标绝对值实现进位，向倒第一说了再见。今天的衡水，经济基础更加扎实，进入了加速起飞的关键时期，蕴含着经济发展的勃勃生机和巨大潜力。

市县财力大幅提升，实现了由保障型财政向发展型财政转变。全部财政收入由59.3亿元增长到163.3亿元，一般公共预算收入由28亿元增长到 88.5亿元，一般公共预算支出由112亿元增长到272亿元。各县市财政收入均超3亿元，6县市突破10亿元。今天的衡水，政府家底更加厚实，人民生活更加殷实，我们能集中更多财力办大事、办群众迫切需要的事。

基础条件显著改善，实现了由要素流出向吸引集聚转变。主城区面积由44平方公里增加到63平方公里。全市新增公路近2000公里，公路网总里程达到 1.27万公里。省级开发区由4个增加到14个，实现了县县有省级开发区。累计引进项目710个，总投资890亿元，各类技术服务4.25万人次。今天的衡水，承载力、吸引力不断增强，城市增值的综合效应加快显现，成为京津冀宜居宜业的新热土。

机遇优势叠加放大，实现了由被动边缘向主动融合转变。衡水发展的重大诉求在顶层设计得到体现，首次进入国家战略层面，被列为京津冀协同发展重要节点城市、国家中小城市综合改革试点和全省综合配套改革试验市，办成了一批多年想办而没有办成的大事要事，政策红利加速释放，后发优势日益凸显，在全国、全省的战略地位显著提升。

精神风貌焕然一新，实现了由封闭保守向创新创业转变。今天的衡水，干部群众的思想观念更加解放，争先进位的信心勇气更足，干事创业、谋大谋新氛围浓厚，夙兴夜寐、激情工作蔚然成风，为创造衡水更加美好的明天，汇集了更加强劲的正能量。

在肯定成绩的同时，我们也清醒地认识到，当前衡水正处在爬坡过坎、转型升级的关键时期，一些深层次矛盾和问题尚未得到根本解决，一些新的困难和挑战又在不断出现。经济发展总量小、水平低，传统行业发展受限，新兴产业培育不足，转型升级任务艰巨。城乡建设相对滞后，城镇规模小、功能差、辐射带动能力不强，美丽乡村建设任务艰巨。深化改革需要突破，对外开放亟待加强。环境保护形势依然严峻，资源环境约束日益加剧，大气、水、土壤污染问题仍然突出，污染治理和生态修复还需付出巨大努力。政府职能转变还不到位，一些地方和部门的服务意识不强、行政效率不高。对这些问题，我们一定高度重视，采取有力有效措施切实加以解决。

各位代表!回顾过去五年，成就令人振奋，经验弥足珍贵。我们深切体会到，推进衡水经济社会又好又快发展，必须始终坚持以习近平总书记系列重要讲话精神和对河北的重要指示为引领，坚定不移贯彻中央大政方针，确保国家和省重大决策部署在衡水落地生根;始终坚持不断解放思想，深化改革开放，大力实施创新驱动发展战略，以创新推动结构调整、促进转型升级、抢占发展制高点，实现发展动能的加快转换;始终坚持强势开局、跨越赶超基调不变，强化危机意识，树立追赶意识，以质量效益为中心，紧抓发展第一要务不放松，努力开辟衡水各项工作新境界;始终坚持打基础、铸优势，保持定力，精准发力，埋头苦干累积发展后劲，负重爬坡厚植发展优势，变隐性优势为显性优势、落后差距为后发优势、比较优势为竞争优势，走差异竞争的发展之路;始终坚持普惠民生，增进人民福祉，财力向民生投放，政策向民生倾斜，着力提升公共服务水平，切实解决群众最关心最现实的利益问题，让发展成果更多更公平惠及全市人民。

各位代表!“十二五”时期我市经济社会发展取得的成就，是省委、省政府和中共衡水市委正确领导的结果，是全市人民艰苦奋斗、锐意进取、共同努力的结果。在此，我代表市人民政府，向全市人民，向人大代表、政协委员，向各民主党派、工商联、无党派人士和人民团体，向驻衡部队、武警官兵和政法干警，向驻衡中省直各单位，向所有关心支持衡水发展的海内外各界朋友，表示崇高的敬意和衷心的感谢!二、“十三五”时期经济社会发展的指导思想和主要任务

“十三五”时期是我市全面建成小康社会的决胜期，是全面深化改革的攻坚期，也是推进绿色崛起、跨越赶超的关键期。当前，衡水已经站上了一个崭新的发展平台，积蓄了向更高层次发展、向更高目标迈进的新动能。肩负全市人民的重托，承载新的历史使命，我们要准确把握新常态带来的趋势性变化和我市所处的历史方位，以一往无前的信心抢抓机遇，以战无不胜的决心迎接挑战，以舍我其谁的责任感和时不我待的紧迫感，夙兴夜寐、激情工作，努力把衡水推上经济更加繁荣、社会更加和谐、生态更加优美、人民更加幸福的新轨道。

“十三五”时期我市经济社会发展的指导思想是：全面贯彻党的十八大和十八届三中、四中、五中全会精神，以邓小平理论、“三个代表”重要思想、科学发展观为指导，深入贯彻落实习近平总书记系列重要讲话精神，高举发展、团结、奋斗的旗帜，按照“五位一体”总体布局和“四个全面”战略布局，坚持发展第一要务，牢固树立和贯彻落实创新、协调、绿色、开放、共享发展理念，加快形成引领经济发展新常态的体制机制和发展方式，坚守发展、生态和民生三条底线，把握协同发展、转型升级、又好又快的工作总基调，按照省委八届十二次全会要求和市委三届七次全会部署，坚持以提高发展质量和效益为中心，继续实施“项目立市、工业强市、三产兴市、农业升级、绿色崛起”发展战略，全面深化改革，提高开放水平，着力推动产业结构深度调整，着力推进新型城镇化，着力建设现代农业强市，着力优化生态环境，着力保障和改善民生，坚决打赢脱贫攻坚战，确保与全国全省同步全面建成小康社会，全力建设经济强市、美丽衡水。

经济强市、美丽衡水，是落实中央和省委、省政府要求，立足衡水实际提出的奋斗目标。建设经济强市，就是要加快赶超步伐，经济总量、质量、效益实现增比进位突破;创新能力不断增强，新技术、新产业、新业态蓬勃发展，初步形成具有衡水特色的现代产业体系;立市立县大项目、大园区、大企业加速培育，每个县市区都要上一个百亿元项目，打造一个主营业务收入超500亿元乃至超千亿元的产业园区，培育几个产值过百亿元的大企业、大集团、大龙头。打造美丽衡水，就是要生态环境持续改善，大气污染治理和生态修复等重点工程见到明显成效，彻底甩掉“全国后十”的黑帽子;城乡面貌明显改观，具有衡水特色的城乡统筹发展路子基本定型;城乡居民收入增速始终高于全省平均水平，期末绝对值达到或接近全省平均水平，人民群众幸福感、获得感不断增强。

综合考虑我市基础条件和发展环境，“十三五”时期主要目标设定为28项，其中预期性指标13.5项，约束性指标14.5项。总体目标就是“一个高于、两个提升、三个提前、一个确保”，即：主要经济指标增速高于全省平均水平;全部财政收入占地区生产总值、一般公共预算收入占全部财政收入比重进一步提升，其中一般公共预算收入年均增长10%以上;地区生产总值、城乡居民收入提前比2010年翻一番，贫困人口和贫困县提前实现脱贫摘帽，地区生产总值年均增长8.5%以上，城乡居民人均可支配收入年均分别增长10%、11%;确保如期全面建成小康社会。未来五年，重点抓好以下七大任务：

(一)持之以恒推进创新发展，加速培育经济发展内生动力。搭建创新平台，培育创新主体，优化创新环境，努力形成大众创业、万众创新的生动局面，使创新真正成为发展的主动力。用好京津创新资源，与京津共建科技园区、创新服务平台，共建技术交易市场、产业技术创新联盟，建设经济技术成果转化基地。加大创新投入，重点针对节能环保、新能源开发、智能装备制造、生物科技等核心关键技术，实施一批重大科技专项，依托六大主导产业创建国家级实验室。强化校企合作、产学研用协同创新。实施苗圃、雏鹰、科技小巨人和“新三板”上市工程，科技型中小企业数量比2015年翻一番以上。实施“百千万”人才兴衡计划，建立“创业导师”工作机制，实施“蓝火计划”衡水专项，壮大创新人才队伍。

(二)持之以恒推进改革发展，增强经济社会发展活力。全面推进国家中小城市改革试点和省级综合配套改革试验市工作，鼓励基层和部门推出“一招鲜”举措，建成全省城乡一体化发展先行区、“四化”同步发展示范区、黑龙港地区快速发展引领区。从供给侧结构性改革发力，实现由低水平供需平衡向高水平供需平衡跃升。推进行政管理体制改革，简政放权，降低行政成本，提高政府效能。推进国有企业分类改革和监管，深化财税体制改革。创新地方金融机构管理体制机制，培育多层次金融机构，开发应用多类别金融产品，加强金融风险管控，满足经济发展需求。深化农村土地制度改革，创新农村集体产权制度，培育新型农业经营主体。推进“五个平台两头+”改革模式，使供销社更好服务“三农”。发挥市场配置资源的决定性作用，推进土地、资本、技术等要素市场建设，完善价格形成机制和市场监管机制。完善党委领导、政府主导、社会协同、公众参与、依法保障的社会治理体制，促进社会治理精细化。

(三)持之以恒推进转型发展，提高经济发展质量和效益。大力推进农业供给侧结构性改革，加快发展以高端设施农业为重点的现代农业园区建设，促进一二三产业协调联动和农业转型升级。实施“中国制造2025”和“互联网+”计划，开展技术和管理改造，努力推动特色产业向主导产业跨越、主导产业向支柱产业跨越，壮大完善由铁路交通工程、功能材料及制品、食品及生物制品、节能环保及新能源、物料输储装备、电力通讯装备六大主导产业和现代农业、现代物流、文化旅游三大产业构建的“6+3”产业体系。改造提升传统优势产业，集中发展电子信息、大健康新医药、高端装备制造等战略性新兴产业，全面提高产品技术、工艺装备、能效环保水平。到“十三五”末，食品及生物制品业主营业务收入超500亿元，功能材料及制品制造业超1000亿元，战略性新兴产业占规模以上工业增加值比重达到20%以上。加快发展现代服务业，推动生产性服务业向专业化和价值链高端延伸，生活性服务业向便利化、精细化和高品质转变。以打造冀中南区域性物流枢纽为目标，建设集多式联运、仓储配送、供应链管理、信息化为一体的现代物流体系。依托衡水湖湿地等生态文化资源，加快体验性、参与性和休闲性项目建设，打造区域性知名旅游城市和生态休闲度假旅游目的地。加快发展会展经济、服务外包等新兴业态。推动传统文化产业转型升级，培育发展新兴文化业态，把文化产业发展成为支柱产业。到“十三五”末，服务业增加值占地区生产总值比重达到45%以上。

(四)持之以恒推进协调发展，全力构筑平衡发展新格局。健全农村基础设施投入长效机制，完善城镇基础设施和公共服务设施网络，构建“一核三轴三区”城镇空间布局。做大做强中心城市，改造提升桃城区、冀州市，加速发展滨湖新区和工业新区，实现市区建成面积100平方公里、湖区水面100平方公里、环湖造林100平方公里、市区人口100万人目标。深化城市管理体制改革，加快城市经济发展步伐。实施县城建设三年攻坚行动，做足“水、绿、净、文”四篇文章，打造宜居宜业特色县城。支持桃城区提升中心城区功能，打造冀中南商贸物流集散中心;支持冀州市打造现代产业新高地和文化旅游目的地;支持枣强县建设智能装备制造基地和裘皮服饰生产基地;支持武邑县打造衡武产业协同发展区;支持深州市建设高档家居、精细化工和优质果品生产加工基地;支持武强县建设现代农业示范区、国际乐器文化产业基地和文化旅游基地;支持饶阳县打造京津优质果蔬产品供应基地和农副产品物流集散基地;支持安平县打造丝网之都和国家级区域物流服务中心;支持故城县打造服装服饰产业聚集区和优质农副产品供应基地;支持景县打造现代装备制造、石化科技新材料、橡塑管业、铁塔产业制造基地;支持阜城县建设衡水东部开放经济试验区、港口经济延伸区、小城镇建设示范区和优质安全农副产品加工配送基地。以产业小镇、明星小镇、特色小镇为模式，扎实推进建制镇建设。以环境美、产业美、精神美、生态美为要求，推进美丽乡村建设和“三区同建”。力争常住人口城镇化率和户籍人口城镇化率分别达到60%、 45%。

(五)持之以恒推进绿色发展，着力优化生态环境。以生态约束倒逼发展转型，以发展转型保障生态改善，打造京津冀生态环境支撑区。落实最严格的环境保护制度，推进大气、水、土壤污染防治行动，加强联防联控和流域共治，确保劣五类水质河流基本恢复使用功能，细颗粒物浓度下降达到省要求。开展气候生态承载量科学评估，加强衡水湖生态保护与修复。加快地下水超采综合治理，统筹实施引水入城入园入村工程。推进造林绿化转型升级和林业产业化经营，力争“十三五”末森林覆盖率提高到33.5%。全面节约和高效循环利用资源，强化约束性指标管理，实行能源和水资源、建设用地等总量和强度“双控制”。大力发展循环经济，打造近零碳排放示范区。加强生态文明制度建设，建立资源环境生态红线制度和资源审计制度，逐步完善生态保护补偿机制和环境信息公开制度。强化环保行政执法和刑事司法联动，实行生态环境损害责任终身追究制度。

(六)持之以恒推进开放发展，构建开放型经济体系。围绕“一枢纽四基地”功能定位，以交通、生态环保、产业三个领域为突破口，精准承接非首都功能疏解，继续实施“三个一百”工程，努力建设京津冀世界级城市群重要节点城市。围绕打通京衡、津衡、石衡、衡港、衡济五大通道，推动一批重大交通基础设施项目建设，构建高铁、城际、轨道、高速、航空等互联互通交通网络。积极融入“一带一路”战略，开展同东盟、南亚、中东、俄罗斯等国家和地区的企业合作。积极承接电子信息、装备制造、文化创意等服务外包项目，建设服务外包合作基地。抓好工业新区海关监管场所、综合保税区、保税物流中心、保税仓库等各类海关特殊监管区域和保税海关场所的申报和建设，创新跨境电商等外贸方式，提高对外贸易规模和质量。推进安平丝网、大营裘皮等国家级外贸转型升级示范基地建设。

(七)持之以恒推进共享发展，持续保障和改善民生。全面落实“六个精准”要求，实施“五个一批”行动计划，确保“十三五”末现行标准下农村贫困人口实现脱贫，贫困县提前摘帽。健全工资增长机制，推行企业工资集体协商制度，落实公务员工资水平政策调整机制、事业单位绩效工资制度。积极推进创业基地建设，重点做好高校毕业生、农业转移人口、城镇困难人员和退役军人就业工作。落实统一的城乡居民基本养老保险制度，推进机关事业单位养老保险制度改革，完善城镇职工、城乡居民基本养老保险制度和城乡居民基本医疗保险制度，实施全民参保计划，探索“医养结合”养老模式。推动农村小学高年级向乡镇集中、初中向重点镇集中、高中和职业教育向县城教育园区集中，普及高中阶段教育，发展职业教育，吸引京津重点高校来我市联合办学，打造京津冀教育名城。深化医疗卫生体制改革，建立覆盖城乡的基本医疗卫生制度和现代医院管理制度。深入开展全民健身运动，打造“国际绿色马拉松”品牌赛事，提升长跑城、健步城、篮球城建设水平。积极应对人口老龄化，完善社会养老服务体系。深入推进平安衡水建设，加强社会治安综合治理。严格落实安全生产责任和管理制度。实施食品药品安全放心工程，确保“舌尖上的安全”。

各位代表!“十三五”宏伟蓝图已经绘就，决胜全面建成小康社会的号角已经吹响。只要我们万众一心、奋力拼搏，艰苦创业、苦干实干，就一定能够开创经济强市、美丽衡水建设的新局面，就一定能够实现全面建成小康社会的奋斗目标，就一定能够再铸衡水新的辉煌!三、2016年主要工作

今年是全面建成小康社会决胜阶段的开局之年，也是推进结构性改革的攻坚之年，做好今年的政府工作十分重要。我们要认真落实党的十八届五中全会、中央扶贫开发工作会议、中央经济工作会议、中央城市工作会议和省委八届十二次全会、全省经济工作会议、全省扶贫开发工作会议、全省城市工作会议，以及市委三届七次全会、全市经济工作会议精神，全面贯彻创新、协调、绿色、开放、共享发展理念，适应引领经济发展新常态，按照宏观政策要稳、产业政策要准、微观政策要活、改革政策要实、社会政策要托底的要求，战略上打好持久战，战术上打好歼灭战，围绕推进供给侧结构性改革，创造性落实好去产能、去库存、去杠杆、降成本、补短板五大任务，最大限度用好政策机遇，推动经济社会又好又快发展。

主要预期目标是：地区生产总值增长8.5%，规模以上工业增加值增长7%以上，全部财政收入增长10%，一般公共预算收入增长12%，固定资产投资增长15%，社会消费品零售总额增长10%，进出口总值及出口总值均增长5%，城乡居民人均可支配收入分别增长10.5%、11.5%，细颗粒物浓度下降 8%，全年空气质量优良天数达到133天。居民消费价格指数、城镇登记失业率、单位生产总值能耗和主要污染物排放等约束性指标均控制在计划指标之内。

工作中重点把握好以下几点：一是着力去产能，推动产业迈向中高端水平。坚决遏制和淘汰没有市场需求的落后产能，坚决处置和关停一批没有竞争能力和盈利能力、污染环境、低效占用宝贵资源，特别是依靠财政“输血”、银行贷款存活的“僵尸企业”。着眼现有产业提档升级，争取上级财税支持、不良资产处置、失业人员再就业和生活保障、专项奖补等政策，加大产业整合重组力度，提升技术装备水平，优化存量产能，做大优势产能，推动产业向高端化、智能化、绿色化方向迈进。二是着力去库存，促进房地产市场健康发展。扎实推进以满足新市民需求为出发点的住房制度改革，打通供需通道，有效释放刚性需求和改善性需求，加快农民工市民化，走具有衡水特色的去库存工作路子。坚持从供需两个方向发力，把握未来房地产市场需求走势，促进房地产业稳定健康发展。三是着力去杠杆，积极拓展金融空间。我市政府债务总体水平不高，在全省占比较小。要抓住国家出台债务置换措施的机遇，提高直接融资比例，降低融资成本，确保融资资金真正用到需要的项目上。做好债务置换、地方融资平台改制、实物资产证券化等工作，处置好存量债务。做大全市金融总量，增加长期项目贷款，多渠道、多元化筹集项目建设资金，向上争取更大信贷资金规模，满足基础设施建设和实体经济发展需求。四是着力降成本，进一步优化企业发展环境。对接国家和省降低制度性交易成本、人工成本、企业税费负担、社会保险费、企业财务成本、电力价格、物流成本等方面的降本增效政策措施，不折不扣抓好落实，设立企业应急转贷资金池，积极主动为企业排忧解难，千方百计降低生产经营成本，让企业“轻装上阵”。五是着力补短板，切实增强发展后劲。正视我市发展不足的市情，把抢抓机遇补短板作为重大战略任务，务求实现大的突破。突出在新型城镇化上补短板，解决城镇化率低、城镇建设管理水平不高、城市经济发展不足等问题。突出在重大基础设施建设上补短板，加紧谋划和实施一批重大交通设施项目，解决城市基础设施和公共服务设施建设滞后等问题。突出在民生事业上补短板，解决公共服务体系不完善，基本公共服务不均衡，教育、文化、医疗卫生、就业和社会保障服务水平不高等问题。突出在脱贫攻坚上补短板，坚决打赢脱贫攻坚战。

(一)全力稳增长，培育发展新动能。稳增长就要抓牢项目不放松。狠抓项目建设。开展重大项目提升年活动，完善“三个二”项目推进机制，实施 “1123”工程，重点抓好市级以上重点项目100项，谋划推进立市立县重大项目100项，新开工亿元以上项目200项，推进市政重大工程30项。加快烨和祥生物玉米纤维、金太阳立体仓库成套装备、华尔特高科技丝网等项目建设，力促舜鑫现代智能物流装备、泰国正大肉鸡全产业链、双诚液态金属等项目开工建设。精准对接国家“7+4”重大工程包，用足用好国家专项建设基金，谋划建设一批新的重大基础设施项目。实施创新驱动战略。顺应新一轮科技革命和产业变革潮流，出台实施新兴产业“双创”三年行动计划，发展众创、众包、众筹等新模式，发挥集众智、汇众力的乘数效应，催生新经济成长。实施高新技术产业三年倍增和科技型中小企业成长计划，年内新增高新技术企业10家、科技型中小企业500家，高新技术产业占规模以上工业增加值比重达到17.5%以上。继续实施现代企业制度建设、企业家素质提升和企业整合重组三大工程，激发企业家创业创新激情，推动企业制度、管理、商业模式创新。加快创新平台和体系建设，每县至少建成一个创新平台。做优做特开发区。出台关于全市省级经济开发区体制机制改革的指导意见，深化开发区体制机制改革，推广政府引导、企业经营、市场运作、多元投入的经营模式，鼓励引导外资、民资和各类社会资本参与开发区的开发运营。坚持高端定位、高标准建设，对开发区现有规划再完善、再提升，特别要积极融入京津冀协同发展、“一带一路”、中东欧合作、“互联网+”等重大战略。

(二)紧盯促协同，确保政策措施落地见效。把京津冀协同发展贯穿到经济社会发展各领域各环节，精准对接，重点突破，努力在区域协同中加速衡水跨越赶超。紧盯京津冀协同发展“十三五”规划，争取一批含金量高的政策措施，推动一批改革创新、先行先试政策在我市试点。编制我市京津冀协同发展规划，制定落实国家、省一系列专项规划的实施方案。突出抓好工业新区和滨湖新区两大承接京津产业转移平台。工业新区要围绕建成千亿园区、争创国家级开发区目标，统筹生产、办公、生活、商业等功能建设，高起点推进产城融合，打造食品产业园、服装产业园、医药产业园和新型功能材料产业园;启动运行“蓝火计划”衡水基地和北京、天津两个众创空间，落实北京秋实食品、红螺食品等项目要件，确保尽快开工建设，努力成为全市科学发展的先行区，转方式、调结构的起步区，改革开放、城乡统筹的试验区，强势开局、跨越赶超的引领区，全市经济社会发展的增长极。争取滨湖新区列入首批承接北京非首都功能疏解的微中心，积极推进京津等地著名医疗机构在衡水湖建设远程康复中心和区域医疗卫生平台，打造京津教育、体育、医疗、健生功能疏解基地。支持各县市区与京津开展多层次协作，共建产业园区。加强与石家庄、沧州等地交流合作，打造石衡沧经济带。

(三)加快转方式，提升产业层次水平。坚持把提高质量效益放在核心位置，在加快发展的同时更好推动发展转型。推进工业产业提质增效。实施“6+5行动计划”，六大主导产业每个产业选择5家重点骨干企业给予点对点支持。实施“1339”工程，努力把功能材料及制品制造业打造为全市的支柱产业。支持企业重组整合，形成一批主业突出、竞争力强的大企业、大集团。支持老白干集团打造百亿元企业。开展制造业升级专项行动，全面实施新一轮技术改造，年内培育省级工业化和信息化深度融合重点企业8家。大力发展高端装备、生物医药等战略性新兴产业，增速要高出地区生产总值3个百分点以上。帮助企业提档升级，新增规模以上工业企业95家。加快发展现代服务业。抓好国家级现代商贸物流中心、衡德综合物流园、新发地农副产品物流园等建设。启动衡水国际会展中心建设，打造区域性、综合性商务会议聚集中心。高标准编制全市旅游业发展总体规划，加快健步路等体验性、休闲性项目建设，衡水湖争创5A级旅游景区，全市接待国内外游客和旅游总收入分别增长20%、15%以上。培育壮大文化产业。推进武强国际乐器、饶阳民族乐器、故城青竹美术画材、武邑硬木雕刻等文化产业集聚区建设，培育一批特色文化产业集群。推动报业、广电、演艺拓展创新，提高文化产品和服务的附加值，引导传统文化企业加强与创意企业、互联网企业的交流合作。加快现代农业强市建设。围绕建设安全食品和优质农产品生产加工配送基地，推动规模农业、法人农业、科技农业提档升级。大力发展新型农业经营主体，新增示范家庭农场 500家，培育市级示范家庭农场100家，力争创建省级示范家庭农场20家、省级示范园区3家，抓好覆盖饶阳县全域的国家级现代农业公园建设。加快建设 “五线、十片”优质蔬菜产品基地，新增设施瓜菜1万亩。构建粮经饲统筹、种养加一体、三次产业深度融合发展的现代农业结构，肉、蛋、奶产量分别达到51万吨、42万吨和33万吨。

(四)打造新引擎，加快新型城镇化建设。推动中心城市扩容升级。优化主城区、建设滨湖新区、提升冀州城区，完善功能区布局，扩展城市发展空间。抓好国家园林城市创建。突出湖城特色，科学规划城市绿地系统，加快道路绿化、庭院绿化、园林绿化，大力推进广场公园建设，建成区绿化覆盖率、绿地率和人均公园绿地面积分别达到36.09%、31.11%和11.28平方米。高标准规划建设城市立体停车场8座。完善城市治理体系，加快海绵城市、绿色节能建设，加强城中村改造和地下管廊建设。以智慧城市建设为抓手，实行数字化、标准化、网格化、精细化管理，构建城市管理新模式。实施市政工程“3个10”计划。106 国道衡水湖段改线、榕花大街改造提升、文化艺术中心、“一河三街”亮化提升等10个已开工项目年内建成投用，石衡高速、大广高速衡水湖连接线、植物园等 10个项目确保开工建设，加快推进衡东物流基地、桃冀轨道交通等10个项目前期工作。围绕把高铁新区打造成具有现代活力的标志性新城区，启动建设高铁站及站前区道路、绿化、管网、广场和停车场。统筹推进县城、小城镇、美丽乡村建设。实施县城建设三年攻坚行动，加快产城教融合，今年所有县城实现集中供水、集中供热或清洁能源供热、污水垃圾达标处理。经济实力较强的县谋划建设总投资不低于50亿元、其他县谋划建设总投资不低于30亿元的城建项目。选择一批优势明显、发展潜力大的镇重点培育，打造一批特色小镇、产业小镇。积极争取撤乡设镇和撤镇设街道办事处。实施美丽乡村建设规划，抓好418个重点村建设。推进多村联建和“三区同建”，抓好村庄撤并整合。着力推进人的城镇化。坚持以人为核心，尊重规律，提高城市发展的持续性、宜居性，把促进有能力在城镇稳定就业的常住人口有序实现市民化作为首要任务，加快户籍制度改革和居住证制度落地，确保常住人口城镇化率和户籍人口城镇化率分别达到50%、35.5%，较上年提高3.4个、2.5个百分点。

(五)强力抓改革，提升对外开放水平。以综合配套改革为统领，加快改革创新步伐，激发经济活力。扎实开展“放管服”工作。实施行政审批瘦身提速行动，规范和完善建设项目并联审批工作，减少审批环节，缩短审批流程。深化商事制度改革，落实“先照后证”，探索“证照分离”“多证合一”，试行电子营业执照。加快行业协会商会与行政机关脱钩，清理规范行政许可中介服务事项。完善政务云平台建设，推进政府大数据应用，提高政府服务能力。深入推进重点领域改革。深化资源要素配置方式改革，提高科研人员成果转化收益分享比例。深化农业农村改革，完善土地承包经营权确权登记工作，规范土地流转。深化财税制度改革，完善财政体制，抓好绩效预算管理改革，全面落实“营改增”任务，推进结构性减税和普遍性降费，开展水资源费改税试点。深化金融领域改革，加快城市商业银行发展，发展股权基金、金融租赁等新业态，规范发展互联网金融。推进养元公司在上交所上市，瑞丰、中科能源等在港交所上市，新增上市企业10家以上。深化公共文化服务体系建设，以县级文化馆、图书馆为中心推进总分馆制，加快基层综合性文化服务中心建设。深化县级公立医院综合改革。完成公务用车制度改革。深化扩大对外开放。积极搭建平台，支持企业参与各类贸易促进活动，开拓国际市场特别是东盟、中东等新兴市场。引导企业在技术、品牌、服务等方面上水平，提升国际竞争力。落实国家和省外贸稳增长政策，在提高贸易便利化水平、清理涉企收费等环节，研究采取实打实的支持举措。

(六)铁腕治污染，切实改善生态环境。坚持以改善环境质量为核心，打好治污攻坚战。铁腕治理大气污染。围绕“治煤、治尘、治气”三大核心任务，实施六大防治工程。实施城中村煤改气工程，对市区环内及环外近郊300多个村实施燃煤改燃气。实施燃煤锅炉淘汰工程，全部淘汰1500台10蒸吨以下燃煤锅炉。实施挥发性有机物治理工程，推进工业企业挥发性有机物污染治理，整治餐饮业油烟、所有涉及喷漆工序的4S店和汽修厂，完不成治理任务的坚决取缔。实施人工干预天气工程，在市区西部建设6个人工干预天气作业点，增加降水量，提升空气质量。实施“互联网+环保”工程，建立全天候、立体化监控指挥平台，提高大气污染防治精准化、精细化、精确化水平。实施机动车新能源替代工程，购置160辆新能源公交车，市区老旧燃油公交车全部替换。加大水、土壤污染防治力度。实施水污染防治工程、工业园区污染治理工程和滏阳河综合整治工程，确保废水达标排放。制定实施土壤污染防治行动计划，实行土壤分区分类管控，综合防治农业面源污染。加快生态修复步伐。采取“节、引、蓄、调、管”等有力措施，推进坑塘河渠连通蓄水、引水灌溉建设，新增地下水压采能力1.18亿立方米。继续推进南水北调，新建12座地表水厂全部投入运行，年引用长江水6200万立方米以上。围绕打造京津生态屏障保护基地，重点抓好林业园区建设、绿美廊道建设、环衡水湖绿化，完成造林15万亩。抓好节能减排。加强工业、交通、建筑和公共机构等重点领域节能，强化政府减排控制。实施排污许可证制度，在企业层面实行排污许可证“一证式”管理，建立企业守法等级评价和黑名单制度。发展循环经济，推动重点工业园区循环化改造。严格环保执法。突出重点区域、重点企业环境监管，完善五级网格化环境监管体系，深入开展环保治理专项行动。强化环评监督管理，促进产业项目合理布局。倡导低碳生活，把环保教育融入到中小学社会实践课，提高全社会环保意识，营造保护环境的良好氛围。

(七)尽心惠民生，办好为民利民实事。以提高人民幸福指数为目标，推进幸福衡水建设，着力抓好六大惠民工程。精准扶贫脱贫工程。围绕脱贫目标精准施策，整合资金解决突出问题，加强就业帮扶拓宽增收渠道，抓好教育帮扶阻断代际传递，提高医保水平减少因病致贫，建强队伍提高能力，层层压实责任，级级传导压力，确保13万贫困人口稳定脱贫。扩大就业工程。促进大学生创业就业，探索构建面向人人的众创空间服务平台。完善就业实名制管理动态办法，加强职业技能培训，认真解决农村转移劳动力、困难群体等就业问题。全年新增城镇就业3.75万人。教育提质工程。继续实施第二期学前教育三年行动计划，建设幼儿园 109所，继续推进省级示范型幼儿园建设。抓好主城区中小学规划建设，扩建小学2所，投入使用新建小学2所，进一步缓解市区中小学班容量过大问题。推动安平、阜城义务教育县域内均衡化试点工作。继续推进“全面改薄”工作，改善农村中小学办学条件。加强实训基地建设，支持衡水学院向应用技术大学转型。社会保障工程。做好城乡养老保险制度衔接，提高企业退休人员养老金、城乡低保、农村五保对象补助水平和社会保险待遇水平。建立统一的城乡居民基本医疗保险制度，加强城乡医保信息化建设和精细化管理，推动城乡居民大病保险规范运行，新型农村合作医疗筹资标准和城镇居民医保补助标准分别提高到人均570元、420 元。实施全面两孩政策。住房改善工程。实施棚户区改造1.2万套，完成农村危房改造2190户。切实抓好房地产去库存，打通商品房与安置房、保障性住房的转换通道，深入开展房地产市场专项整治，全面落实促进房地产市场健康发展的政策措施。完善社区物业管理服务，探索自治管理新模式。文体繁荣工程。办好全市文艺调演、全民阅读等系列文化活动，推动文化下乡、文化惠民工程和农村电影放映工作，丰富群众文化生活。推进“两馆一站”免费开放。推进以篮球城、长跑城、健步城为重点的体育名城建设，继续办好衡水湖国际马拉松赛，积极组织开展丰富多彩的全民健身活动。扎实推进精神文明建设，培育践行社会主义核心价值观，发扬传承中华传统文化，努力培育向上向善、诚信互助的社会新风尚。

(八)扎实防风险，维护社会和谐稳定。加强和创新社会管理，化解和消除各类矛盾与风险，最大限度增加和谐因素。加强社会治安综合治理。构建立体化社会治安防控体系，提高警务科技应用水平，完善警务指挥机制，推行社会信用代码制度。推进智慧交通建设，推行“e居卡”流动人口管理模式。加强社会矛盾排查化解。完善社会矛盾源头预防和调处化解机制，加强改进信访和调解工作，有效预防和化解矛盾纠纷。完善市县群众工作中心建设，打造“一站式”服务平台。加强乡村便民服务站点标准化建设，扩大服务范围，真正成为解决群众诉求的“终点站”。加强金融风险防控。完善金融监管体制，及时发现处置重大金融风险及突发事件，依法打击非法集资等金融违法行为，坚决守住不发生系统性和区域性金融风险的底线。加强安全生产和食品药品监督管理。深入开展安全生产专项整治，加大 “打非治违”力度，坚决遏制重特大安全事故发生。加强食品药品全过程监管，推进智能食品药品安全信息化和检验检测能力建设，建设市食品药品检验检测中心，抓好食品药品安全县创建。

加强国防教育和国防后备力量建设，促进军民融合深度发展，巩固军政军民团结。充分发挥工会、共青团、妇联等人民团体桥梁纽带作用。继续做好新闻出版广电、民族宗教、外事侨务、防震减灾、邮政通信、史志档案、援疆、妇女儿童、老龄、残疾人等工作。四、切实加强政府自身建设

各位代表!发展新起点，人民新期待，要求我们必须全力推进政府自身建设，以更加开阔的视野、更加昂扬的斗志、更加务实的作风，更好地担当起推动衡水新一轮跨越发展的历史重任。

大力解放思想，建设改革政府。坚持高标准、高要求推动“十三五”发展，努力促进各项工作在全省增比进位。创新理念、创新方法，坚决破除各种思维定势和体制障碍，谋划推出一批具有重大牵引作用的改革发展举措，进一步开拓衡水发展新境界。把夙兴夜寐、激情工作作为常态，把马上就办、办就办好作为工作要求，以“人一之我十之、人十之我百之”的精神，勇于突破发展中遇到的复杂问题和深层次矛盾，在每个岗位上创造新业绩、展示新形象、体现新作为。

践行服务宗旨，建设为民政府。始终把人民利益放在第一位，努力把群众的期待变成我们的行动，把群众的希望变成生活的现实。坚持保基本、兜底线、补短板，把新增财力更多用于民生，抓好为民办实事项目，不断加大公共服务产品供给。积极开展接访、走访、回访活动，真诚倾听群众呼声，真实反映群众愿望，真情关心群众疾苦。

持续改进作风，建设有为政府。主动作为、靠前服务，强化抓重点、抓关键的工作机制，重大事项层层签订责任书，确保各项工作部署落地生根。充分发扬政企互动的好传统，旗帜鲜明支持企业经营发展，理直气壮维护企业合法权益，不遗余力为企业排忧解难。巩固“三严三实”专题教育成果，开展机关作风整顿，严肃查处各种损害群众利益的行为。加强政府督查工作，健全激励问责机制，有效治理慢作为、不作为、乱作为，做到政令畅通。

坚持依法行政，建设法治政府。加强法治政府建设，做好“七五”普法，深化政务公开，加强行政复议和行政应诉工作，严格规范行政执法行为，进一步提高政府执行力和公信力。自觉接受人大法律监督、工作监督和政协民主监督，提高人大代表建议和政协委员提案的办理质量，充分听取各民主党派、工商联、无党派人士以及各人民团体的意见和建议，认真接受新闻舆论和人民群众监督。

严守红线底线，建设廉洁政府。坚持把纪律和规矩挺在前面，认真贯彻《中国共产党廉洁自律准则》和《中国共产党纪律处分条例》，切实履行党风廉政建设主体责任，全面落实“一岗双责”。加强行政监察和审计监督。严格执行中央八项规定，持续加大“四风”整治力度，树立为民、务实、清廉政府形象。

各位代表!建设经济强市、美丽衡水的美好愿景鼓舞着我们，全市人民对幸福生活的向往激励着我们!让我们紧密团结在以习近平同志为总书记的党中央周围，在省委、省政府和中共衡水市委的坚强领导下，解放思想、抢抓机遇、奋发作为、跨越赶超，为实现“十三五”规划目标任务、全面建成小康社会，谱写衡水发展新篇章而努力奋斗!
